# Cellular Biogenetic Law and Its Distortion by Protein Interactions: A Possible Unified Framework for Cancer Biology and Regenerative Medicine

**DOI:** 10.3390/ijms231911486

**Published:** 2022-09-29

**Authors:** Alexander E. Vinogradov, Olga V. Anatskaya

**Affiliations:** Institute of Cytology, Russian Academy of Sciences, 194064 St. Petersburg, Russia

**Keywords:** cell differentiation, gene phylostratigraphy, gene expression, interactome, embryonic stem cells, induced pluripotent stem cells, recapitulation law, Heckel’s law, humans, whole genome duplication, evolutionary medicine

## Abstract

The biogenetic law (recapitulation law) states that ontogenesis recapitulates phylogenesis. However, this law can be distorted by the modification of development. We showed the recapitulation of phylogenesis during the differentiation of various cell types, using a meta-analysis of human single-cell transcriptomes, with the control for cell cycle activity and the improved phylostratigraphy (gene dating). The multipotent progenitors, differentiated from pluripotent embryonic stem cells (ESC), showed the downregulation of unicellular (UC) genes and the upregulation of multicellular (MC) genes, but only in the case of those originating up to the Euteleostomi (bony vertebrates). This picture strikingly resembles the evolutionary profile of regulatory gene expansion due to gene duplication in the human genome. The recapitulation of phylogenesis in the induced pluripotent stem cells (iPSC) during their differentiation resembles the ESC pattern. The unipotent erythroblasts differentiating into erythrocytes showed the downregulation of UC genes and the upregulation of MC genes originating after the Euteleostomi. The MC interactome neighborhood of a protein encoded by a UC gene reverses the gene expression pattern. The functional analysis showed that the evolved environment of the UC proteins is typical for protein modifiers and signaling-related proteins. Besides a fundamental aspect, this approach can provide a unified framework for cancer biology and regenerative/rejuvenation medicine because oncogenesis can be defined as an atavistic reversal to a UC state, while regeneration and rejuvenation require an ontogenetic reversal.

## 1. Introduction

The biogenetic law (recapitulation law, von Baer’s law, Heckel’s law) states that ontogenesis recapitulates phylogenesis [1,2,3]. This law assumes a ‘terminal addition’ when recently evolved features are added at the last stages of development, nearing the adult state [4]. However, recapitulation can be distorted by evolutionary modifications appearing at any developmental stage, especially by embryonic adaptations [1,5]. For a long time, this has been a debated topic; however, recently, the concept of ontogenetic recapitulation has acquired new support from molecular and anatomical studies [1,3,4]. Currently, the biogenetic law is becoming especially important because of the atavistic theory of oncogenesis, which suggests that cancer is an evolutionary reversal to a unicellular state [6,7,8,9,10].

The genes of unicellular (UC) origin are overexpressed in cancer tissues, whereas the genes appearing in the multicellular (MC) evolutionary stages are downregulated [11,12,13]. The human interactome (global protein interaction network) contains giant clusters, one of which is strongly enriched with the genes of UC origin and corresponding functions, while the others are enriched with the genes of MC origin and their functions, which suggests the existence of an MC/UC contrast in cellular networks [14]. The genes downregulated with human aging are enriched in the UC cluster, whereas the upregulated genes are overrepresented in the MC cluster. The clusters show denser interactions within them than between them; therefore, they can serve as attractors (stable states of dynamic systems) for cellular programs. Importantly, the UC cluster has a higher inside/outside connection ratio compared with the MC clusters (i.e., it is denser), which suggests a stronger attractor effect and may explain why the cells of MC organisms are prone to oncogenesis (reversal to the UC state) [14].

The UC cluster is upregulated in human cancers, which was shown in the case of the single-cell transcriptomes of various cancer types with the control of the cell cycle activity [15]. The expression of genes involved in the cell cycle is correlated with the expression of UC genes, even if the overlapped genes are removed; therefore, the control of the cell cycle activity is necessary for the demonstration of evolutionary reversal in cancer cells. These data suggest that oncogenesis is not just the alteration of a few genes but the switch to ancient unicellular programs. Therefore, the comparison of cancer cells with the organisms belonging to the UC evolutionary stage may help us to elucidate the etiology of diseases and aging and even to suggest possible remedies. For instance, certain unicellular-specific drugs can be applied for the treatment of cancer [16,17]. Certain other diseases can also be understood as a result of evolutionary reversal [4]. The gene expression shift towards earlier evolutionary stages was also observed in the polyploidization of somatic cells, which can be considered as the activation of the cell emergency reserve under stressful conditions [18].

The biogenetic principle may also be important for regenerative/rejuvenation medicine, which is intrinsically intertwined with cancer biology. The main contradiction of multicellularity (MCM) is that between the cellular and organismal levels [14]. The cell pluripotency and proliferative potential are vital for the healthy development and longevity of MC organisms if held in check. In this case, the activity of the UC level promotes the organism’s vitality. In contrast, unchecked unicellularity results in oncogenesis when the cells tend to behave as independent evolutionary units [8,16,19]. In this case, the activity of the UC level undermines the organism’s vitality. The main problem for the application of stem cell technology in regenerative medicine is the question of how to avoid oncogenesis [20,21]. These two opposite forces—promotion vs. suppression of the MC organism’s vitality by the activity of the UC level—are encapsulated by the term ‘MCM’. As an example of the UC/MC contrast in cellular networks, the total pluripotency signature (PluriNet) is enriched in the UC giant cluster, whereas the genes controlling pluripotency (the KEGG pathway) are enriched in the MC cluster [14].

The atavistic theory of cancer entails that the study of the biogenetic law at the cellular level is especially important. Before the study of a pathology, it is necessary to know the basics of the normal development, i.e., how the evolution is recapitulated in cell differentiation, which constitutes an essential part of ontogenesis (and whose reversal is an essential part of oncogenesis). This knowledge can also be helpful for regenerative medicine and rejuvenation (or prolongation of the healthy lifespan) because the reversal of development may be associated with the reversal of expression to more ancient genes and cellular programs. This process may be similar to oncogenesis but should include differences, ensuring safe reversal. Thus, the cellular-level study of recapitulation can extend the importance of the biogenetic law from a purely academic field to the practical dimension and help researchers to build a unified framework for cancer biology and regenerative/rejuvenation medicine.

Recently, the appearance of single-cell transcriptomes has made it possible for researchers to investigate the biogenetic law at the cellular level. Here, we study the cellular recapitulation of phylogenesis with an emphasis on the UC–MC evolutionary transition. This work presents a meta-analysis of human single-cell transcriptomes in the pluripotent embryonic stem cells (ESC), more differentiated cells (multipotent progenitors and unipotent erythroblasts), embryonic cells during zygotic cleavage, and induced pluripotent stem cells (iPSC). We estimated the relative effects of ontogenetic recapitulation and development modernization, assessing the impact of the evolutionary origin of tested genes and the genes encoding for interactomes of the proteins encoded by the tested genes on the expression of the tested genes during cell differentiation.

Our approach is based on the concept that the modernization of development can be performed by the interaction of the proteins encoded by older genes with more recent ones. To uncover the pure recapitulation effects, we controlled for the cell cycle activity. This was necessary because the earlier embryonic cells have a higher cell cycle activity compared with more differentiated cells, and the higher cell cycle activity is associated with the upregulation of UC genes [15]. This connection could distort the pure recapitulation effects if studied without the correction for the cell cycle activity.

## 2. Results

### 2.1. The Proof of Concept

**Figure 1:**
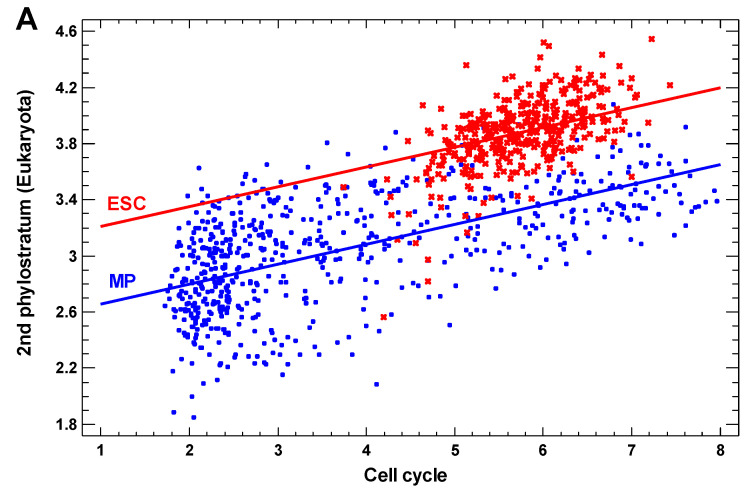

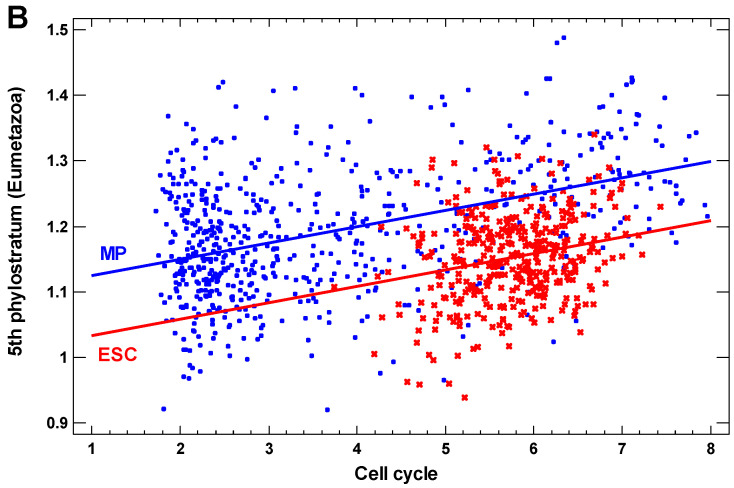
Regression lines of the genes belonging to different phylostrata on the cell cycle signature in the single-cell transcriptomes of multipotent progenitors (MP) (blue) and pluripotent embryonic stem cells (ESC) (red). The mean expression of the genes belonging to a phylostratum is plotted versus the mean expression of cell cycle signature genes (with individual cells as separate points). (**A**) The 2nd phylostratum (unicellular Eukaryota); (**B**) the 5th phylostratum (Eumetazoa). For the difference between intercepts, (**A**) *p* < 10^−103^ and (**B**) *p* < 10^−41^. The transcriptomes are from GSE75748 (‘cell type’ dataset).

We analyzed the transcript levels (henceforth called “expression” for the sake of brevity) of the genes originating at different evolutionary stages (phylostrata) in the single-cell transcriptomes of human cells, which differ in the state of cell differentiation. In the first example, the pluripotent embryonic stem cells (ESC) were compared with the more differentiated multipotent progenitors (MP). As the control for the cell cycle activity, we used the regression lines of the expression of the tested genes on the expression of the cell cycle genes, as previously described [15]. The genes originating in UC phylostrata showed a lower regression line in the MP as compared with the ESC, whereas the genes from the MC phylostrata showed a higher line (Figure 1; Supplementary Figures S1–S17).

**Figure 2:**
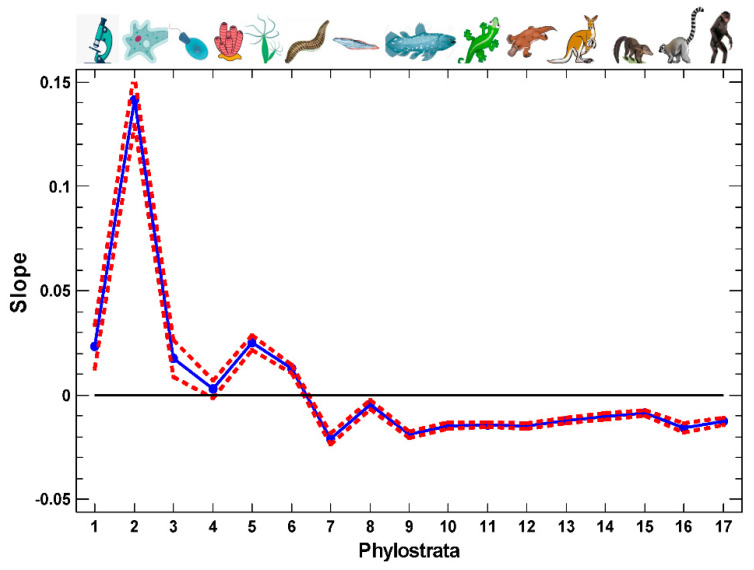
Evolutionary profile of the slope of the regression lines of the expression of genes belonging to different phylostrata on the cell cycle signature in the single-cell transcriptomes, with confidence intervals (*p* = 0.05). The regression lines are shown in Figure 1 and Supplementary Figures S1–S17. The transcriptomes are from GSE75748 (‘cell type’ dataset). Phylostrata: 1—cellular organisms (Prokaryota); 2—Eukaryota; 3—Opisthokonta; 4—Metazoa; 5—Eumetazoa; 6—Bilateria; 7—Chordata; 8—Vertebrata; 9—Euteleostomi; 10—Tetrapoda; 11—Amniota; 12—Mammalia; 13—Theria; 14—Eutheria; 15—Boreoeutheria; 16—Primates; 17—Hominidae. The pictures at the top show recent organisms corresponding to the phyletic branching used for human gene dating.

Importantly, in both cell types, the expression of UC-origin genes correlates with the expression of cell cycle genes (Figure 1). In the MC phylostrata, this correlation sharply decreases, while in the post-Bilateria phylostrata, it becomes negative (Figure 2), but it also requires correction. The negative correlation of the genes from the later phylostrata is understandable because these genes are mostly involved in differentiation and tissue-specific functions (while the UC-origin genes are involved in housekeeping and the cell cycle functions), which are usually associated with the suppression of the cell cycle activity.

Moreover, the ESC show a higher expression of cell cycle genes as compared with the MP. These facts justify the correction for the cell cycle activity. Otherwise, the effect of the evolutionary gene origin on the ESC–MP difference in the gene expression may be distorted by the higher cell cycle activity in the ESC. For this correction, we used the difference in the intercepts between the regression lines for the MP and ESC at equal slopes (see Materials and Methods). By extrapolation, this can be interpreted as the difference in the expression between the MP and ESC at zero cell cycle activity.

**Figure 3:**
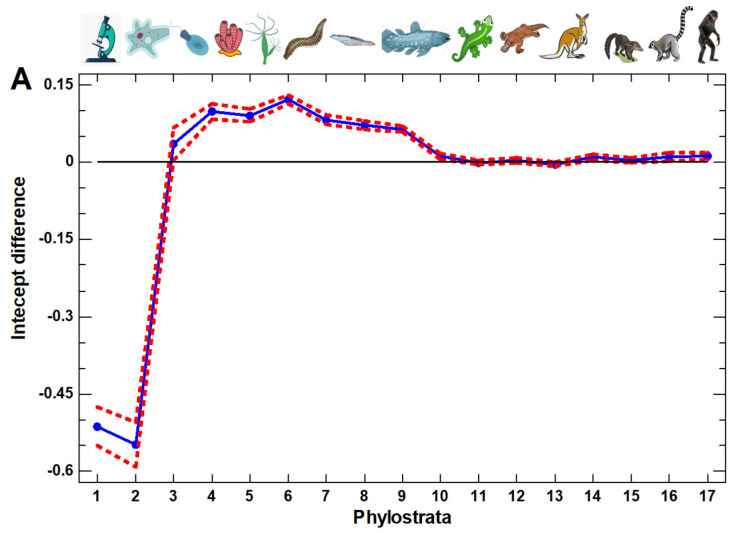

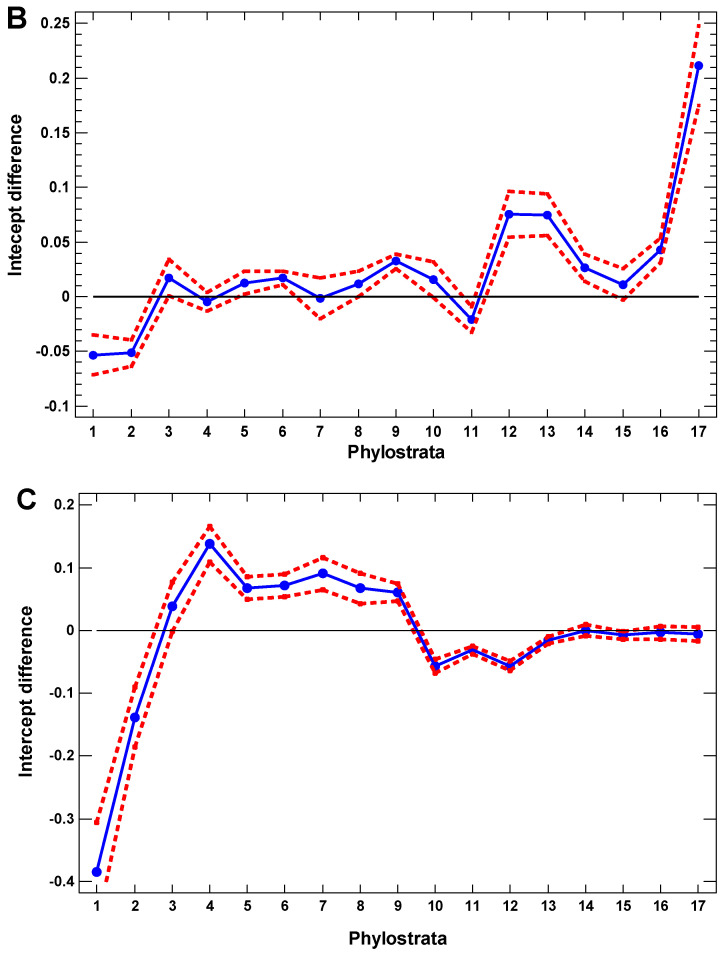
Evolutionary profiles: the differences in the intercepts between the regression lines of the expression of genes belonging to different phylostrata on the cell cycle signature in the single-cell transcriptomes (regression lines as in Figure 1) for the different cell types, with confidence intervals (*p* = 0.05). (**A**) Multipotent progenitors (differentiated from ESC) vs. ESC (GSE75748, ‘cell type’ dataset). (**B**) Erythrocytes (differentiated from erythroblasts) vs. erythroblasts (GSE123899). (**C**) Hepatocyte-like cells (differentiated from iPSC) vs. iPSC (GSE90749). Phylostrata: 1—cellular organisms (Prokaryota); 2—Eukaryota; 3—Opisthokonta; 4—Metazoa; 5—Eumetazoa; 6—Bilateria; 7—Chordata; 8—Vertebrata; 9—Euteleostomi; 10—Tetrapoda; 11—Amniota; 12—Mammalia; 13—Theria; 14—Eutheria; 15—Boreoeutheria; 16—Primates; 17—Hominidae. The pictures at the top show recent organisms corresponding to the phyletic branching used for human gene dating.

For the whole picture across total phylogenesis, we plotted the MP–ESC differences in the intercepts for all the phylostrata (Figure 3A). There are three phases in the evolutionary profile of ESC-to-MP differentiation. The genes that originated in the UC evolutionary stage (the first two phylostrata) are downregulated in the MP as compared with the ESC. Then, at the third phylostratum, there is a sharp transition to the second phase. The difference in the intercepts changes sign, indicating the upregulation of genes originating in the third (and later) phylostrata in the MP as compared with the ESC. The third phylostratum is Opisthokonta (represented by the recent colonial Choanoflagellata), which can be considered as the last unicellulars or first multicellulars, depending on the viewpoint. The second phase of the evolutionary profile (the upregulation in the MP) continues up to the 9th phylostratum (Euteleostomi, bony vertebrates). Beginning from the 10th phylostratum (Tetrapoda: amphibians, reptiles, birds, and mammals), any difference disappeared, which indicated the third phase (the absence of recapitulation).

Thus, the MP–ESC comparison demonstrates that ontogenesis, at the cellular level (reflected in the ESC-to-MP cell differentiation), recapitulates phylogenesis in a phase-like manner, with a sharp UC/MC contrast, but only up to the Euteleostomi. A similar three-phase picture, with a sharp UC/MC contrast at the Opisthokonta and the termination of the recapitulation after the Euteleostomi, can be seen during the 4 days of the ESC culturing, demonstrating the process of differentiation (Supplementary Figures S18 and S19).

The ESC were represented by two cell lines (H1, H9) behaving similarly, whereas the MP were represented by five cell lines, and it is the consolidated picture that is shown in Figure 3A. Taken separately, the MP cell lines show a certain variation, but the three-phase pattern generally remains (Supplementary Figures S20 and S21). The only difference in the pattern of the UC–MC transition was observed in the neural progenitors (NPC) (Supplementary Figure S20A). In the NPC, the genes originating in the third phylostratum (Opisthokonta) show a lower expression in the MP compared with the ESC, and the UC–MC transition is thus delayed to the fourth phylostratum (Metazoa, recent sponges). This difference can arise because the nervous system is of a later evolutionary origin [22]. After the 9th phylostratum (Euteleostomi), there is also some limited variation. The NPC and the endothelial cells (EC) show a slightly higher (but consistent in the adjacent phylostrata) expression compared with the ESC, i.e., a continued recapitulation of phylogenesis (Supplementary Figure S20A,B). At the same time, the foreskin fibroblasts (HFF), trophoblast-like cells (TB) and endoderm derivatives (DEC) show a slightly (but consistently) lower expression, which can be interpreted as a small distortion of the recapitulation (Supplementary Figure S21A–C).

The multipotent progenitors (MP) are not completely differentiated cells. For the later stages, we studied the differentiation of the unipotent erythroblasts that are precursors of erythrocytes (Figure 3B). The erythrocytes are probably one of the most strongly differentiated cell types, which ultimately lose their ability for replication and even transcription. In the differentiating erythroblasts, the first phase transition is the same (UC–MC), but with a more complicated picture after that stage (Figure 3B). Importantly, in contrast to the ESC–MP differentiation, the differentiating erythroblasts show a pronounced recapitulation in the genes originating *after* the Euteleostomi, with the strongest effect in the last phylostratum (Hominidae). Thus, the recapitulation during cell differentiation was observed for the whole evolutionary range from the unicellulars to hominids (albeit, for the later evolutionary stages, only in the terminally differentiated cells).

### 2.2. Artificial Ontogenetic Reversal

The induced pluripotent stem cells (iPSC) are the result of artificial ontogenetic reversal [20]. The evolutionary profile of their differentiation is qualitatively similar to the differentiation of the ESC (Figure 3C). However, in the range of 10–12 phylostrata, there is a consistent downregulation in the differentiated cells as compared with the initial iPSC. This observation indicates a distortion of recapitulation. The two other iPSC examples show a similar violation in this phylostratic area, albeit less pronounced (Supplementary Figure S22A,B). However, a similar distortion was observed in HFF, TB, and DEC, differing from ESC (Supplementary Figure S21A–C). Therefore, this distortion may simply indicate a variation within the general recapitulation pattern during the differentiation of pluripotent cells.

### 2.3. Ab Ovo

**Figure 4:**
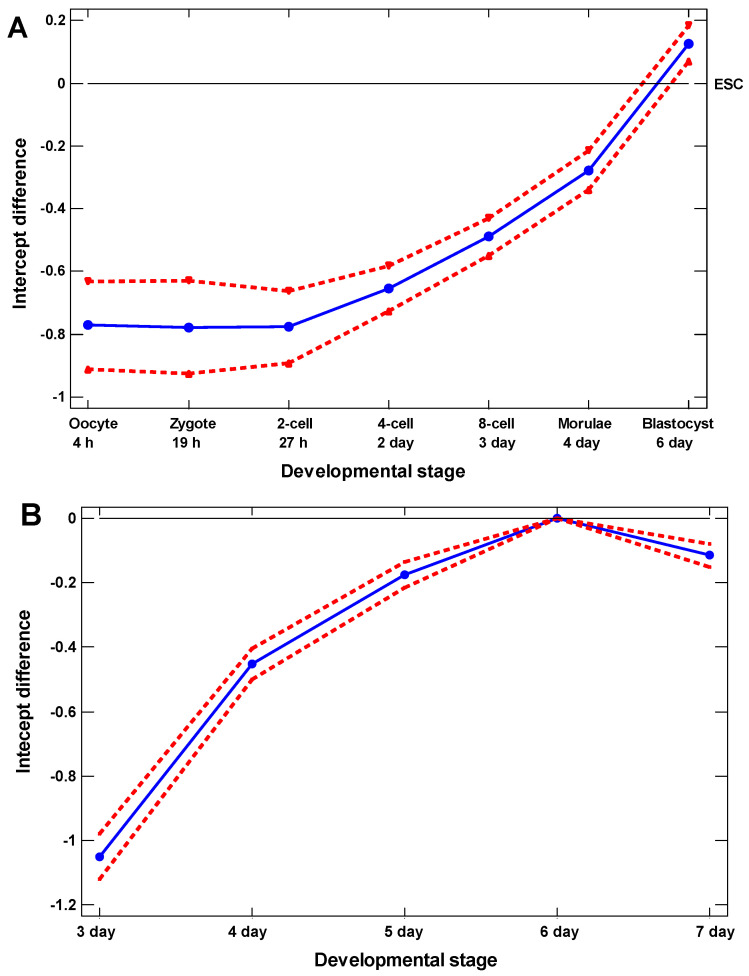
Ontogenetic profile: the difference in the intercepts between the regression lines of the expression of unicellular-origin genes (1–2 phylostrata) on the cell cycle signature in the single-cell transcriptomes from early embryonic development (with confidence intervals, *p* = 0.05). (**A**) Embryonic cells vs. ESC (GSE36552). (**B**) Embryonic cells vs. embryonic cells at day 6 (E-MTAB-3929).

To reveal the earliest appearance of cellular ontogenetic recapitulation, we studied the zygotic cleavage. At first glance, it may be expected that the strongest expression of the UC genes will take place in the UC ontogenetic stage, i.e., in the oocyte or zygote. But this is not so. The highest upregulation of the UC genes was observed in the hatching blastocyst on the 6th day after fertilization (Figure 4). It is known that the ESC exist in the inner cell mass of the human blastocyst from 4th to 7th day after fertilization, and they disappear after the 7th day [23]. Thus, the ESC seem to be very close to the strongest recapitulation of the UC stage, albeit that the upregulation of UC genes is slightly lower in the cultured ESC as compared with the 6-day blastocyst (Figure 4A).

### 2.4. Regulatory Gene Groups

**Figure 5:**
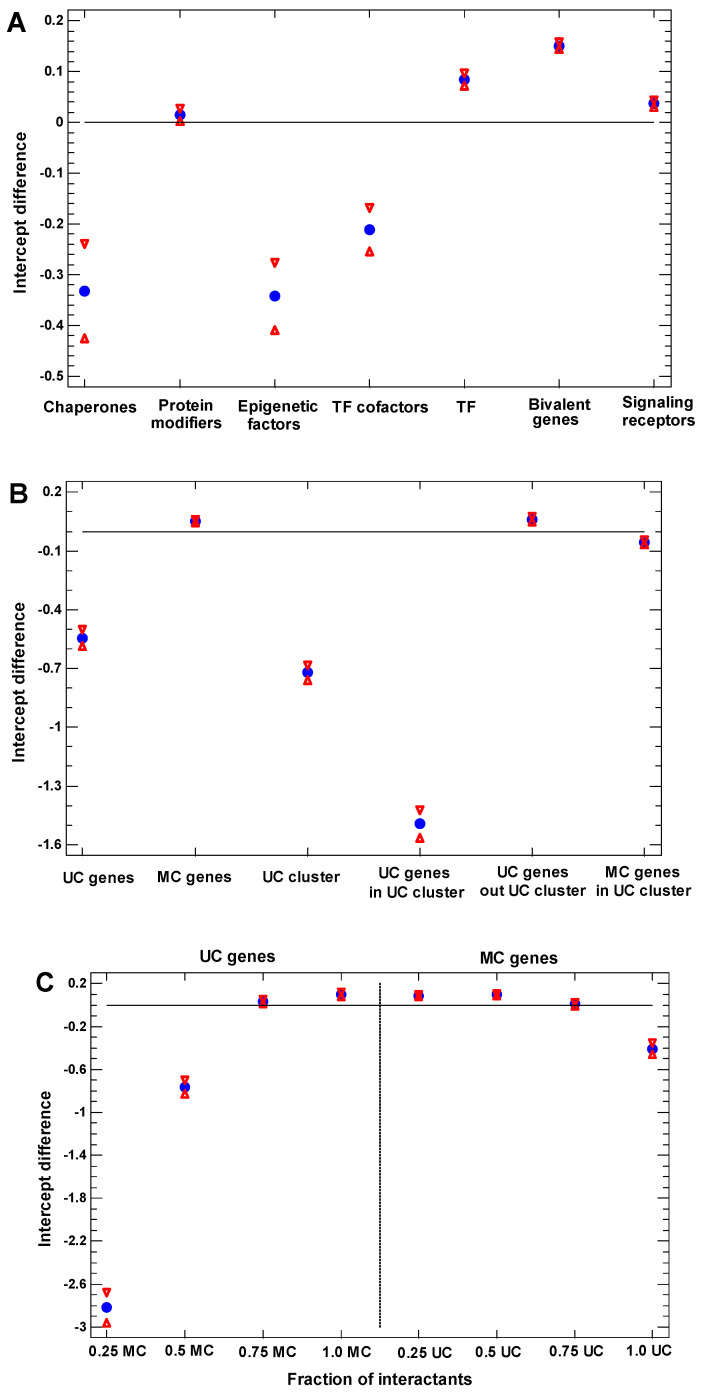
The difference in the intercepts between the regression lines of the expression of different gene groups on the cell cycle signature in the single-cell transcriptomes of MP vs. ESC (GSE75748, ‘cell type’ dataset), with confidence intervals (*p* = 0.05). (**A**) Different regulatory gene groups. (**B**) UC genes, MC genes, and UC giant cluster genes. (**C**) UC and MC genes with different fractions of MC or UC proteins in the one-step interactome neighborhood of their proteins (e.g., ‘0.25 MC’ means 0.25 or a lesser fraction of the MC proteins in the neighborhood of a UC protein). The blue circles show the intercept values, the red triangles show their confidence intervals.

The ESC-to-MP differentiation was chosen for the functional analysis (as it provides the clearest recapitulation pattern of the UC–MC evolutionary transition). Controlling for the cell cycle activity, we studied the expression of regulatory gene groups, whose expansion in the human genome was studied previously using the same phylostratigraphic dating [24]. The chaperones, epigenetic factors, and cofactors of the transcription factors (TF) are downregulated in MP (compared with ESC), whereas the protein modifiers, TF, bivalent genes, and signaling receptors are upregulated in MP (Figure 5A).

### 2.5. The Strength of Old and New Ties

In light of the suggestion that the modernization of development, which distorts recapitulation, can be fulfilled by the interaction of proteins encoded by older genes with more recent genes, we studied the dependence of the gene expression on the evolutionary age of genes encoding for the interactants of proteins encoded by the tested genes. The effect of the interactome proved to be considerable. Thus, albeit that the genes of MC origin are upregulated in the MP (compared with ESC), the MC genes inside the UC giant interactome cluster are downregulated (Figure 5B). For the UC genes, this effect is even more striking. The UC genes inside the UC cluster are much more downregulated in MP (compared with ESC) than the total UC genes, whereas the UC genes outside the UC cluster become even upregulated in MP (instead of being downregulated), thus behaving similarly to the total MC genes (Figure 5B).

At the level of direct (one-step) interactions, we studied the effect of the gradual increase in the MC fraction in the neighborhood of proteins encoded by the tested genes. With the increase in the MC fraction in the one-step neighborhood of a UC protein, the encoding UC gene showed a gradual transition from downregulation to upregulation in MP (compared with ESC) (Figure 5C). With the decrease in the MC fraction in the one-step neighborhood of an MC protein, the encoding MC gene showed a gradual transition from upregulation to downregulation in MP, albeit that this effect of sign changing was weaker than it was in the case of UC genes in the high-MC environment (Figure 5C).

### 2.6. Functional Analysis of the Proteins in Different One-Step Interactome Neighborhoods

**Figure 6:**
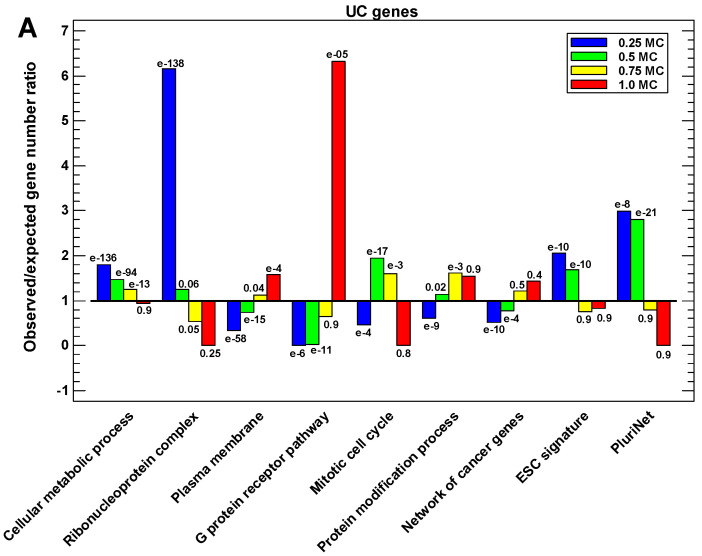

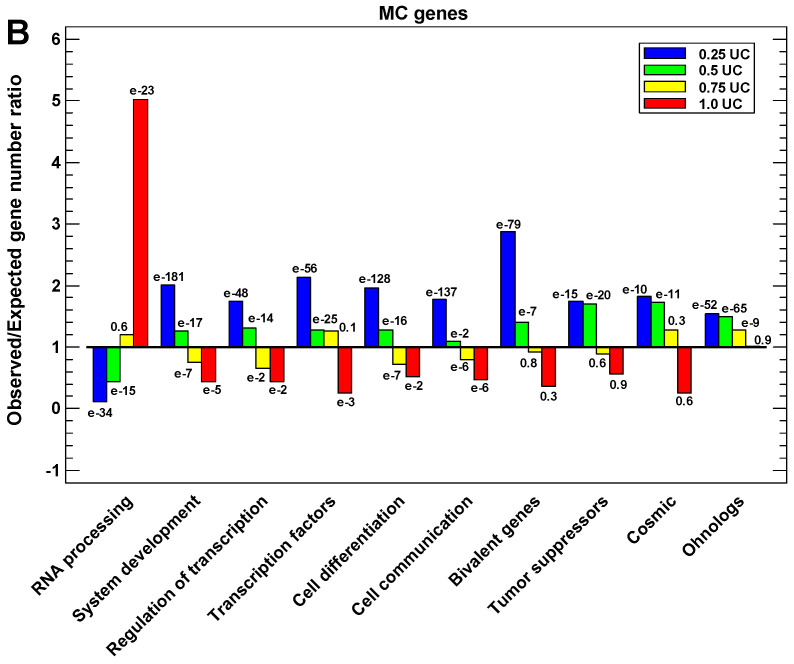
The functional enrichment of the UC and MC proteins with different fractions of MC or UC proteins in their one-step interactome neighborhood. (**A**) UC proteins in the MC environment. (**B**) MC proteins in the UC environment. The significance is either for enrichment (if observed/expected > 1) or for underrepresentation (if O/E < 1).

We studied the functions of the UC and MC proteins differing in terms of the MC fraction in their one-step interactome neighborhoods. For the UC proteins, the conservative UC environment (i.e., a low fraction of MC proteins in the neighborhood) is maintained for the proteins involved in cell metabolism, translation, ribonucleoprotein complexes, and pluripotency signatures (Figure 6A; Supplementary Tables S1–S8). The evolved environment of UC proteins (high fraction of MC proteins in the neighborhood) is observed mostly in the membrane and includes functions related to signaling (Figure 6A; Supplementary Tables S9–S16). The same outcome is observed for protein modifiers (Figure 6A). Importantly, the evolved MC environment is also found in the network of cancer proteins (Figure 6A).

For the MC proteins, the neighborhood with the high UC fraction is observed for the proteins related to RNA processing (Figure 6B; Supplementary Tables S17–S24). The environment with a low UC fraction is observed for the proteins related to development, cell differentiation, cell communication, the regulation of transcription, and transcription factors (Figure 6B; Supplementary Tables S25–S32). The bivalent genes, ohnologs, tumor suppressors, and ‘cosmic’ genes (whose mutations are found in cancer cells) also show a stepwise enrichment with the increase in the MC fraction in the interactome of their proteins (Figure 6B; Supplementary Tables S20, S24, S28, and S32).

## 3. Discussion

### 3.1. Cellular Biogenetic Law

We demonstrated the ontogenetic recapitulation of phylogenesis at the cellular level. The highest upregulation of UC genes was observed not in the single-cell oocyte or zygote but in the hatching blastocyst (about the 6th day after fertilization). This may appear to be a distortion of the biogenetic law, but it only supports it because this observation can be explained by the maternal mRNAs in the zygote. Because of the maternal mRNAs, the oocyte or zygote does not correspond to the UC evolutionary stage but presents a product of the MC organism. Probably, only in the hatching blastocyst does the maternal-to-zygotic transition (MZT) cause the complete decay of maternal mRNAs [25], and the blastocyst transcriptome becomes of a purely zygotic origin. This ontogenetic stage (containing about ten cells) is the strongest recapitulation of the UC evolutionary stage. The upregulation of UC genes in the hatching blastocyst is only slightly higher than in the cultured embryonic stem cells (ESC). Notably, the cultured ESC were initially taken from only the hatching blastocyst [23].

During the differentiation of the pluripotent ESC into multipotent progenitors (MP), the downregulation of UC genes and the upregulation of MC genes take place, albeit only those MC genes that originate up to the Euteleostomi (bony vertebrates). This picture strikingly resembles the evolutionary profile of regulatory gene expansion due to gene duplication in the human genome, which shows a similar decay after the Euteleostomi [24]. The upregulation of the regulatory gene groups also resembles the evolutionary profile of these groups’ expansions. The chaperones, epigenetic factors, and cofactors of transcription factors (TF) are upregulated in the ESC, whereas the protein modifiers, TF, bivalent genes, and signaling receptors are upregulated in the MP.

The only exception is the protein modifiers. In the human genome, the chaperones, epigenetic factors, TF cofactor, and protein modifiers expanded at the UC evolutionary stage, whereas the TF, bivalent genes, and signaling receptors mostly expanded at the MC stages [24]. The exception of the protein modifiers is probably related to the fact that they were adopted for the MC regulation in the course of evolution. Therefore, they became upregulated in the more differentiated cells (MP vs. ESC), where the MC genes are generally upregulated. Similarly, the protein modifiers, which firstly expanded in the genomes of prokaryotes, as the main prokaryotic regulatory level, were adopted in the UC eukaryotes to play the role of epigenetic factors, thereby antecedenting the expansion of TF [24]. For instance, histone modifiers, HATs and HDACs, acetylate and deacetylate thousands of other proteins besides histones [26]. Thus, the recapitulation pattern of the expression of regulatory gene groups in the course of ESC-to-MP differentiation, in general, coincides with the evolutionary course of the expansion of these gene groups in the human genome due to gene duplication (except for protein modifiers), providing additional support for the cellular biogenetic law.

The Euteleostomi evolutionary stage, in which the recapitulation during ESC–MP differentiation is completed, is close to the clade where the vertebrate phylotypic stage is most pronounced [5,27]. A phylotypic stage is a developmental stage, where the embryos of different species belonging to a clade most strongly resemble each other [1,28]. The similarity in the earlier ontogenetic stages is distorted by embryonic adaptations, in the later stages—by terminal additions in the course of clade diversification [5,28]. In the ontogenesis, the phylotypic stage is close to the onset of organogenesis, and the differentiation of MP from ESC is necessary for organogenesis [29,30,31]. The recapitulation of the later evolutionary stages can be observed during the differentiation of the unipotent erythroblasts, where the genes originating at the more recent phylostrata (up to the Hominidae) were upregulated. This differentiation corresponds to the maintenance of definitive tissues.

### 3.2. Modification of Development

The modification of development distorts the recapitulation law. This process is manifested in (and probably caused by) the interactome of proteins encoded by the genes under consideration. The most striking effect for the MC environment is that on the expression of UC genes. There is a stepwise reduction in the downregulation of UC genes in MP (compared with ESC) depending on the MC fraction in the one-step interactome of the UC proteins. Moreover, in the environment with a fraction of MC proteins of about 3/4 or higher, even the upregulation of UC genes takes place. Similarly, the MC genes encoding for proteins in the environment with a UC fraction above 3/4 are downregulated instead of being upregulated. Genes work in the form of proteins, which in turn act as participants of protein interaction networks. It is reasonable to suggest that, after the protein interactions were rewired, the expression of the encoding genes become adapted to the new conditions, in which the encoded proteins found themselves in the rewired interactome. This means that an evolutionary change may begin with a change in the protein sequence (causing changes in the protein interactions) followed by the adjustment of the coding gene expression.

The evolved environment of the UC proteins (i.e., a high fraction of MC proteins in the interactome of a UC protein) includes functions related to signaling, which are mostly performed by protein modifiers. This fact can explain why protein modifiers are upregulated in the more differentiated cells (MP vs. ESC), albeit that their expansion in the human genome took place at the UC evolutionary stage [24]. The signaling is involved in intercellular communications, whose role drastically increases in the multicellulars. The signaling should be performed swiftly, and this can be better achieved by protein modification as compared with changes in the transcription. The evolved MC environment is also found in the network of cancer proteins, which indicates that the control of oncogenesis is the prerogative of the MC level.

For the MC proteins, the environment with a high UC fraction was observed in the proteins related to RNA processing. The environment with a low UC fraction was observed in the proteins related to development, cell differentiation, cell communication, and regulation of transcription. The bivalent genes, which enable rapid switching between cellular programs [32], also show a stepwise enrichment with the decrease in the UC fraction in the interactomes of their proteins. A similar picture was observed for the tumor suppressor and ‘cosmic’ genes (whose mutations were found in cancer cells). Notably, ohnologs (genes retained in duplicates after whole genome duplication) also show a stepwise growth with the decrease in the UC fraction in their interactome environment. Ohnologs are most strongly involved in both the regulatory levels of MC organisms, the nucleome and the nervous system [33].

### 3.3. A Unified Framework for Cancer Biology and Regenerative Medicine

Besides their importance for evolutionary and developmental biology, studies of the cellular biogenetic law can provide a unified framework for cancer biology and regenerative/rejuvenation medicine. The Cancer Genome Project revealed a multitude and great diversity of somatic mutations in cancer cells [34]. In addition, a large number of epigenomic alterations were uncovered [35,36]. These unexpected results raised concerns with respect to the classic ‘somatic mutation theory’ of oncogenesis, which assumes that cancer is caused by the alteration of a few oncogenes, and stimulated interest in the more systemic explanations [34,37,38]. One of the most prominent systemic concepts is the atavistic theory, suggesting that cancer arises because of MC cell reversal to a UC state [6,7,8,9,10]. Similarly, the regeneration/rejuvenation requires a reversal to a younger organism state, which, in accordance with the recapitulation law, may resemble earlier evolutionary stages.

Regeneration is very strongly and paradoxically intertwined with both phylogeny and oncogenesis. The regenerative ability is higher in simpler organisms [39,40,41]. Moreover, in highly regenerative animals (such as salamanders and frogs), regenerative processes can revert malignant cells back to a physiological state [39]. In humans, the regenerative ability is stronger in earlier development, when it can be associated with anticancer activity. Thus, the microenvironment of human embryonic stem cells was reported to suppress the tumorigenic phenotype of aggressive cancer cells [42]. At the same time, the application of stem cell technology for the purpose of regeneration is hindered by the oncogenic potential of stem cells [20,21]. The cellular biogenetic law and its normal (evolution-acquired) distortion by the modification of development may offer a systemic framework for disentangling this knot of intertwined and controversial phenomena.

The genes work not separately but as parts of cellular programs, and these programs were formed in the course of evolution. Probably, they were createdby the addition of extra layers to cellular networks, because the human interactome shows a core-to-periphery evolutionary growth [14], which was accompanied by network rewiring, mixing novel and ancient genes and causing the distortion of the biogenetic law. Before the study of a pathology, it is necessary to obtain a clear picture of normal recapitulation (accompanied by the evolution-acquired modification of development). The deviation from the normal recapitulation can elucidate the etiology of pathological conditions.

Because the regenerative ability is higher among simpler organisms, the controlled activation of earlier evolutionary programs in humans may facilitate injury healing and rejuvenation. ‘Controlled’ is a keyword here, because the danger of oncogenesis is the main problem concerning stem cell usage for regeneration. Probably, healthy regeneration should involve the ontogenetic reversal to a younger state without the phylogenetic reversal to a unicellular state. The search for critical differences between healthy ontogenetic reversal and pathological phylogenetic reversal could benefit from a phylostratigraphic framework representing the history of cellular network building. “*Everything is the way it is because it got that way*” [43] (i.e., everything is explained by its history). The biogenetic law linking development and evolution might offer a central concept for systemic analyses.

The evolutionary approach is also important because many biomedical problems are studied using the model organisms (e.g., rodents, zebrafish, fruit flies, and nematodes). Notably, cancer appeared in the evolution as early as the basal eumetazoans (it was found in hydra and corrals) [19]. Our understanding of the different evolutionary trajectories of model organisms coupled with their recapitulation in ontogenesis is necessary for the correct translation of obtained results to humans.

## 4. Materials and Methods

The human single-cell transcriptomes were acquired from Gene Expression Omnibus [44] and BioStudies [45]. The databases were GSE75748 (two datasets: ‘cell type’ and ‘time course’) [46], GSE123899 [47], GSE90749 (two datasets: ‘hepatocyte-like’ and ‘white adipocytes’) (unpublished), GSE36552 [48], E-MTAB-3929 [49], and GSE81252 [50]. The cell types are indicated in the figure legends (with dataset identifiers).

The control for cell cycle activity was conducted as previously described [15]. Briefly, the data were normalized using the ‘limma’ software implemented in the R package using the ‘quantile’ normalization method [51]. The normalized transcript levels of the genes belonging to a tested gene group (e.g., the genes from a phylostratum) were averaged for each gene group in each cell transcriptome. The limma provides logtransformation. After gene group averaging, the means were back-transformed. We analyzed the regression of the mean of a tested gene group on the mean of the cell cycle signature (the genes from the GO category GO:0000278, ‘mitotic cell cycle’), with the transcriptomes of individual cells taken as separate points. In the text, the transcript level is called “expression” for the sake of brevity. To compare the two regression lines (e.g., MP vs. ESC), we used the difference in the intercepts between these regression lines (at equal slopes), with the corresponding statistical significance. The analyses were performed using the Statgraphics Centurion XVIII package.

As a first approximation, we used the linear model because it enables the strict comparison of the regression lines (with the determination of the statistical significance of the intercept difference between the lines). The comparison of intercepts for nonlinear curves is pointless. Moreover, the linear model grasps the overwhelming part of the variance of the dependent variable explained by the nonlinear model (>90%). For instance, the linear model for the ESC in Figure 1A explained 33.6% of the variance (r-squared coefficient), while the 2-order polynomial model explained 35.9%. (The higher-order polynomial members are not significant.) In other words, linear model represents 94% of the nonlinear model. For the MP in Figure 1A, the r-squared values are 34.7% and 35.5%, respectively. Here, the linear model represents 98% of the nonlinear model. For the ESC in Figure 1B, the r-squared values are 6.5% and 6.6%, respectively. Here, linear model represents 98% of the nonlinear model. For the MP in Figure 1B, the r-squared values are 18.9% and 19.7%. Here, the linear model represents 96% of the nonlinear model.

The evolutionary stratification of human genes (phylostratigraphy, or gene dating) was acquired from [24], where the problems of different gene dating results were discussed. Here, we used shallow phylostratigraphy, which is based on the strict gene orthology obtained using the best reciprocal hits with the accurate Smith–Waterman algorithm. (In contrast, deep phylostratigraphy includes in-paralogs, thus providing the dating of whole gene families.)

The human protein interactions were acquired from the STRING database [52]. We selected the interactions with a top-half confidence (>0.5), which is slightly higher than the default confidence used by the STRING server (>0.4).

The genes encoding for the proteins belonging to the UC and MC giant clusters of the human interactome (used in Figure 5B) were acquired from [14]. For the determination of the fractions of UC- and MC-origin proteins in the one-step interactome neighborhood of a protein (used in Figure 5C and Figure 6), the interactants of this protein were taken from the STRING. Phylostratic gene dating was applied to the genes encoding for these interactants. Then, the fractions of the UC- and MC-origin genes were calculated for this gene set.

The functional over- and under-representation analysis was performed as previously described [53]. For each gene ontology (GO) category, we collected all its subcategories using GO directed acyclic graphs (DAG), and a gene was regarded as belonging to a given category if it was mapped to any of its subcategories. This is necessary because, for instance, only one gene is mapped to the ‘protein modification process’ (GO:0036211) directly, whereas 2500+ genes can be mapped to this process using the GO DAG (because protein modifiers are distributed among specific protein modification processes). The molecular pathways were acquired from the NCBI BioSystems. A redundancy of this resource, which constitutes a most complete compendium of the pathways from different databases, was removed by uniting the entries with identical gene sets.

To this pathways compendium, we added the following gene signatures: the Molecular Signatures Database (MSigDB) [54], tumor suppressor genes from the TSG database [55], genes from the Catalogue of Somatic Mutations in Cancer (COSMIC) [56], human transcription factors from [57] and AnimalTFDB [58], bivalent genes from [32], and genes from the OHNOLOGS database [59]. As the pluripotency signatures, we used PluriNet from MSigDB and the set of genes upregulated in the ESC vs. differentiated cells observed in at least three independent studies [60].

The hypergeometric distribution of probability (implemented in the R environment) was used for the determination of the statistical significance of the ratio of observed to expected numbers of genes belonging to a GO category/pathway in a tested gene sample. The expected number was calculated on the basis of the number of category/pathway genes in the total gene dataset (assuming a random gene distribution across categories/pathways). After the determination of the enriched categories/pathways, the statistical significance of the enrichment was corrected for multiple tests, according to [61].

## Data Availability

The data underlying this article are available in the article and its online Supplementary Materials.

## Supplementary Materials

The following supporting information can be downloaded at: https://www.mdpi.com/article/10.3390/ijms231911486/s1.
