# Supplementary material for: Cellular Biogenetic Law and Its Distortion by Protein Interactions: A Possible Unified Framework for Cancer Biology and Regenerative Medicine
Source: Int J Mol Sci. 2022 Sep 29;23(19):11486. doi: 10.3390/ijms231911486 (PMC9570048; doi:10.3390/ijms231911486)
Supplement: Supplementary file 1 [file ijms-23-11486-s001.zip › SupFigs_S1-S17.pdf]

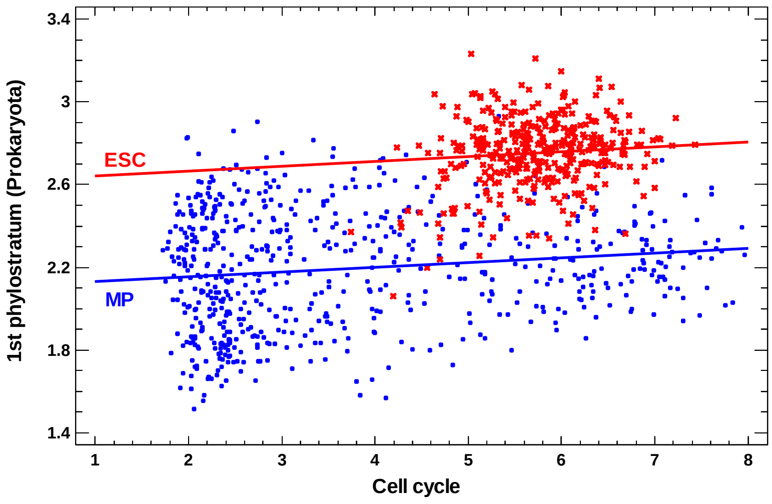

Figure S1. Regression lines of expression of genes belonging to 1st phylostratum (cellular organisms, Prokaryota) on expression of cell-cycle signature in the single-cell transcriptomes of multipotent progenitors (MP) (blue) and pluripotent embryonic stem cells (ESC) (red). For difference between intercepts: $p<10^{-118}$.

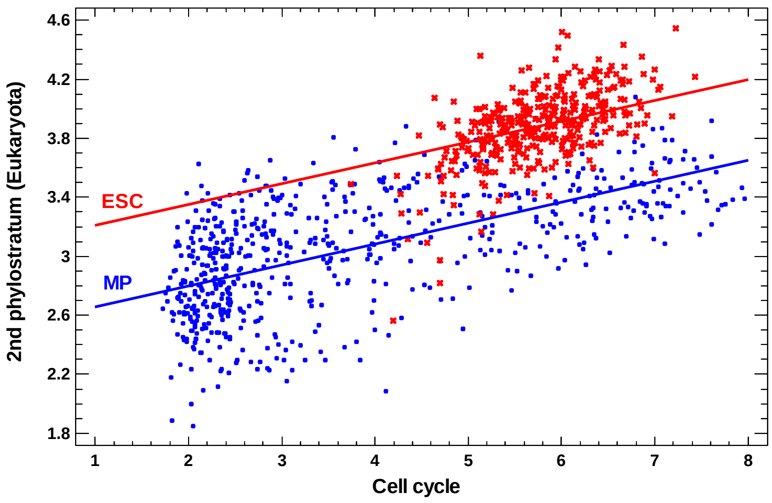

Figure S2. Regression lines of expression of genes belonging to 2nd phylostratum (unicellular Eukaryota) on expression of cell-cycle signature in the single-cell transcriptomes of multipotent progenitors (MP) (blue) and pluripotent embryonic stem cells (ESC) (red). For difference between intercepts: $p<10^{-103}$.

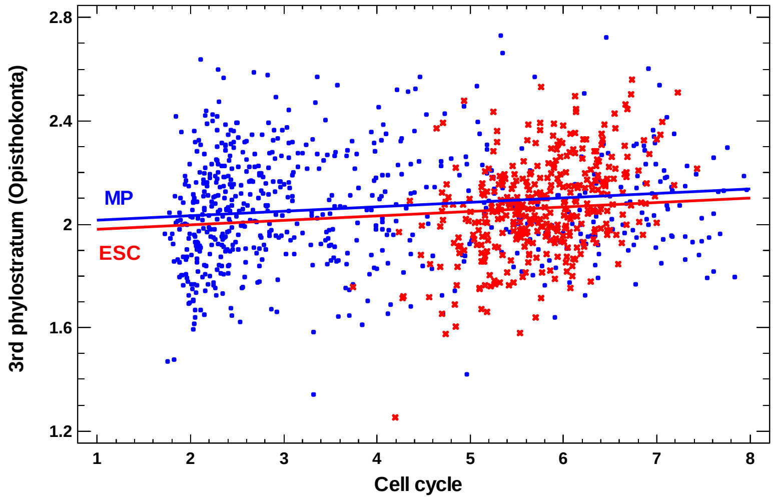

Figure S3. Regression lines of expression of genes belonging to 3rd phylostratum (Opisthokonta) on expression of cell-cycle signature in the single-cell transcriptomes of multipotent progenitors (MP) (blue) and pluripotent embryonic stem cells (ESC) (red). For difference between intercepts: $p<0.03$.

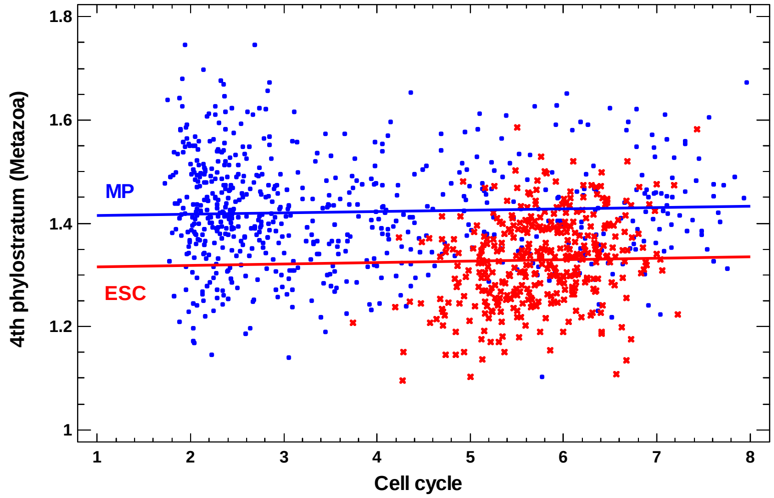

Figure S4. Regression lines of expression of genes belonging to 4th phylostratum (Metazoa) on expression of cell-cycle signature in the single-cell transcriptomes of multipotent progenitors (MP) (blue) and pluripotent embryonic stem cells (ESC) (red). For difference between intercepts: $p<10^{-34}$.

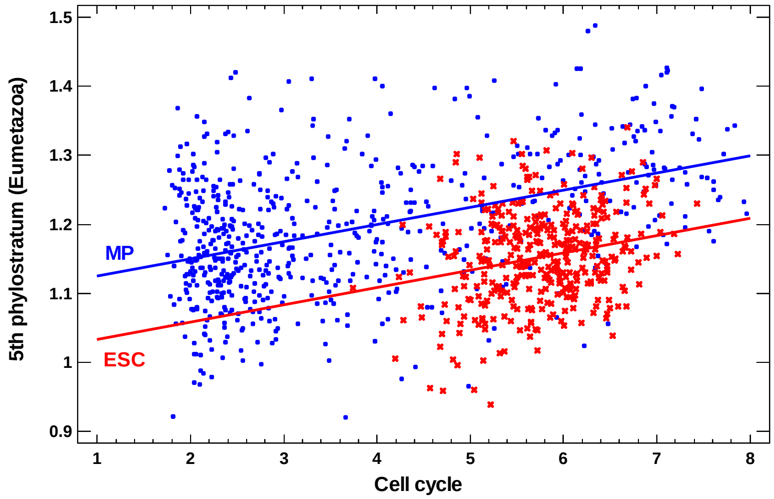

Figure S5. Regression lines of expression of genes belonging to 5th phylostratum (Eumetazoa) on expression of cell-cycle signature in the single-cell transcriptomes of multipotent progenitors (MP) (blue) and pluripotent embryonic stem cells (ESC) (red). For difference between intercepts: $p<10^{-41}$.

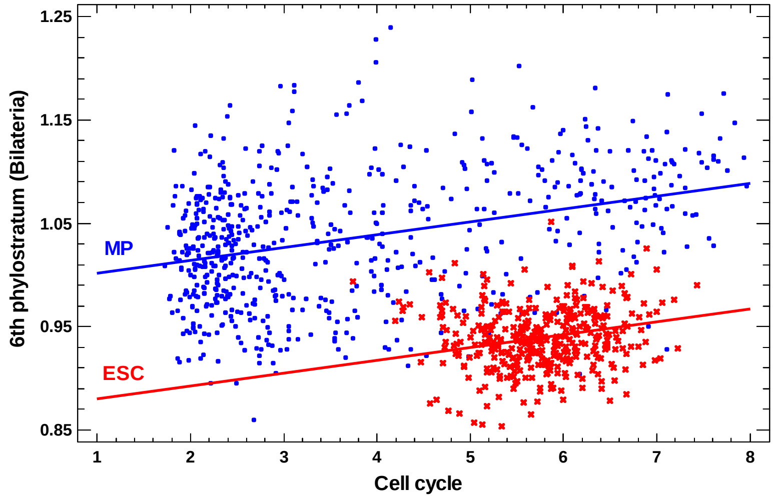

Figure S6. Regression lines of expression of genes belonging to 6th phylostratum (Bilateria) on expression of cell-cycle signature in the single-cell transcriptomes of multipotent progenitors (MP) (blue) and pluripotent embryonic stem cells (ESC) (red). For difference between intercepts: $p<10^{-143}$.

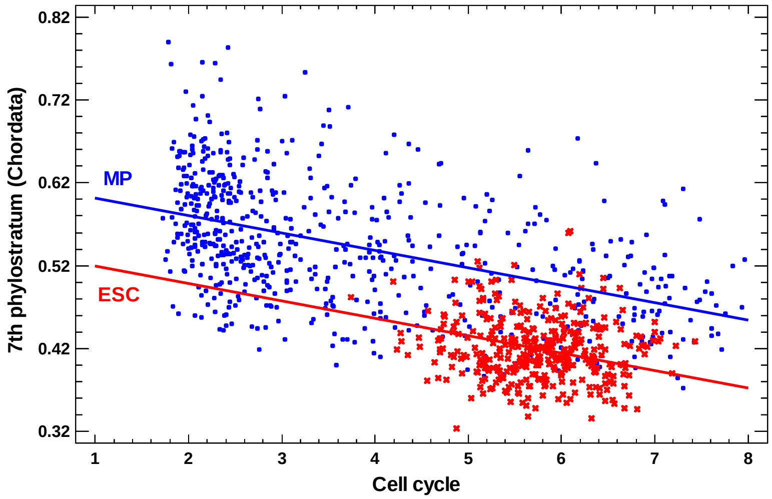

Figure S7. Regression lines of expression of genes belonging to 7th phylostratum (Chordata) on expression of cell-cycle signature in the single-cell transcriptomes of multipotent progenitors (MP) (blue) and pluripotent embryonic stem cells (ESC) (red). For difference between intercepts: $p<10^{-67}$.

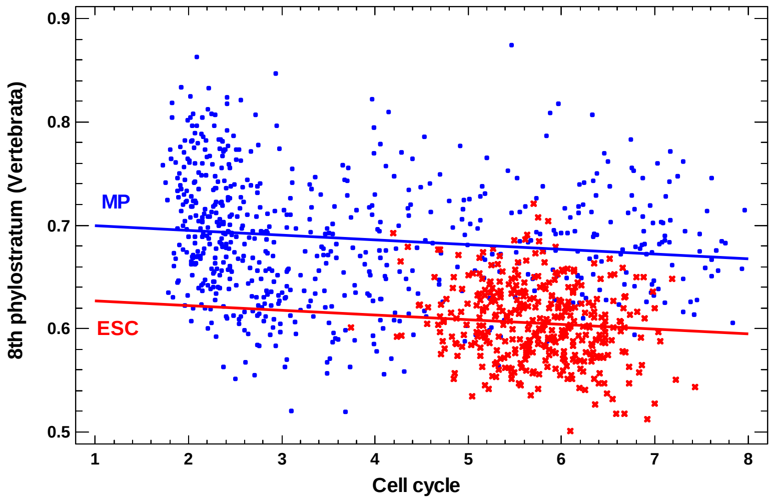

Figure S8. Regression lines of expression of genes belonging to 8th phylostratum (Vertebrata) on expression of cell-cycle signature in the single-cell transcriptomes of multipotent progenitors (MP) (blue) and pluripotent embryonic stem cells (ESC) (red). For difference between intercepts: $p<10^{-60}$.

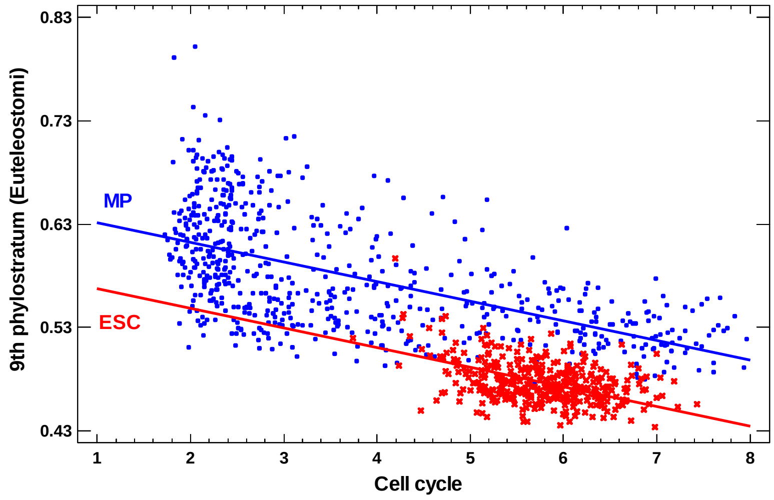

Figure S9. Regression lines of expression of genes belonging to 9th phylostratum (Euteleostomi) on expression of cell-cycle signature in the single-cell transcriptomes of multipotent progenitors (MP) (blue) and pluripotent embryonic stem cells (ESC) (red). For difference between intercepts: $p<10^{-82}$.

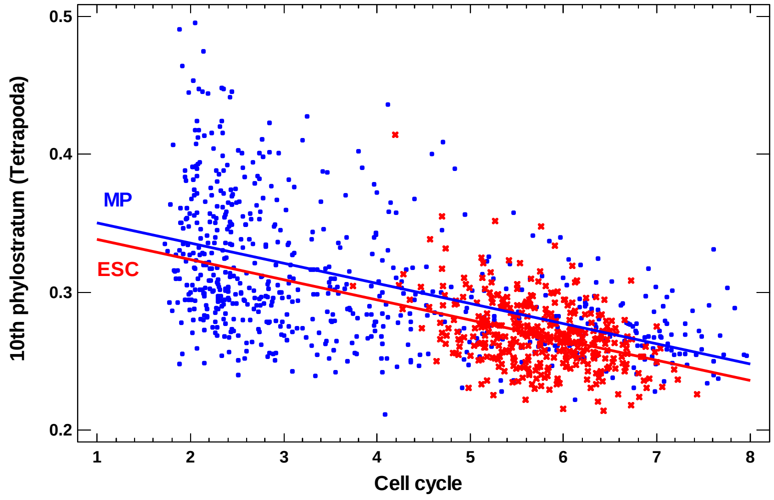

Figure S10. Regression lines of expression of genes belonging to 10th phylostratum (Tetrapoda) on expression of cell-cycle signature in the single-cell transcriptomes of multipotent progenitors (MP) (blue) and pluripotent embryonic stem cells (ESC) (red). For difference between intercepts: $p<10^{-4}$.

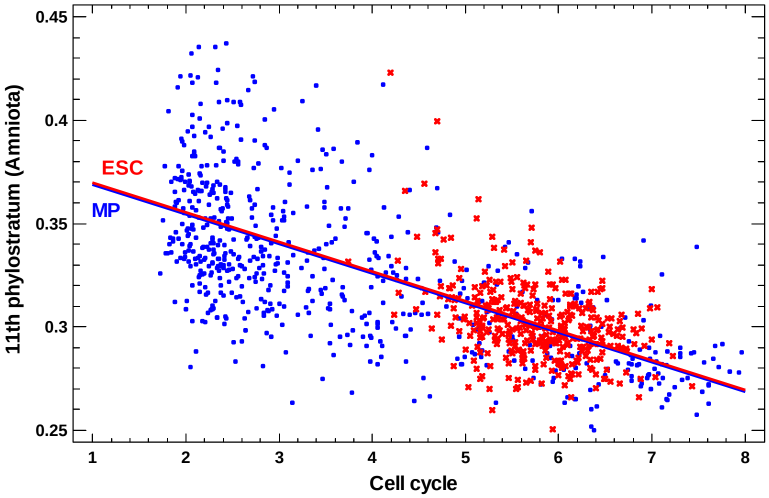

Figure S11. Regression lines of expression of genes belonging to 11th phylostratum (Amniota) on expression of cell-cycle signature in the single-cell transcriptomes of multipotent progenitors (MP) (blue) and pluripotent embryonic stem cells (ESC) (red). For difference between intercepts: $p>0.6$.

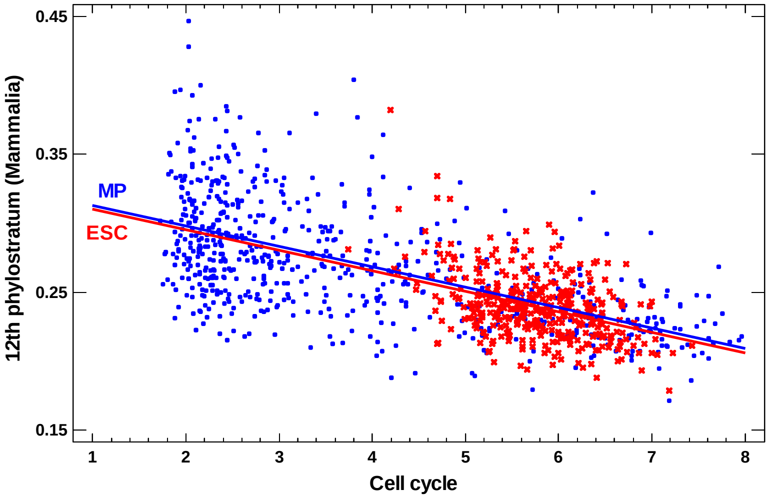

Figure S12. Regression lines of expression of genes belonging to 12th phylostratum (Mammalia) on expression of cell-cycle signature in the single-cell transcriptomes of multipotent progenitors (MP) (blue) and pluripotent embryonic stem cells (ESC) (red). For difference between intercepts: $p>0.2$.

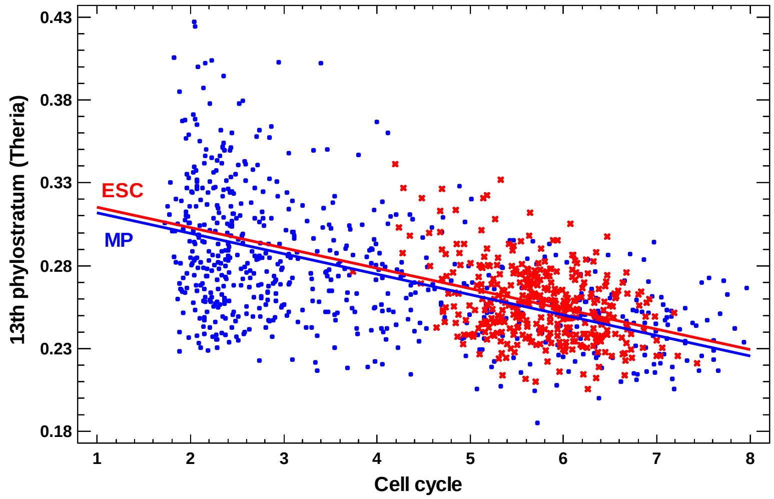

Figure S13. Regression lines of expression of genes belonging to 13th phylostratum (Theria) on expression of cell-cycle signature in the single-cell transcriptomes of multipotent progenitors (MP) (blue) and pluripotent embryonic stem cells (ESC) (red). For difference between intercepts: $p>0.1$.

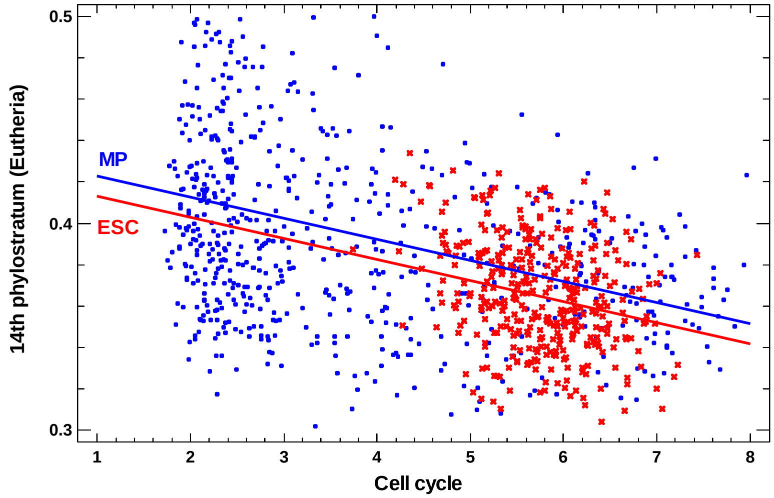

Figure S14. Regression lines of expression of genes belonging to 14th phylostratum (Eutheria) on expression of cell-cycle signature in the single-cell transcriptomes of multipotent progenitors (MP) (blue) and pluripotent embryonic stem cells (ESC) (red). For difference between intercepts: $p<0.01$.

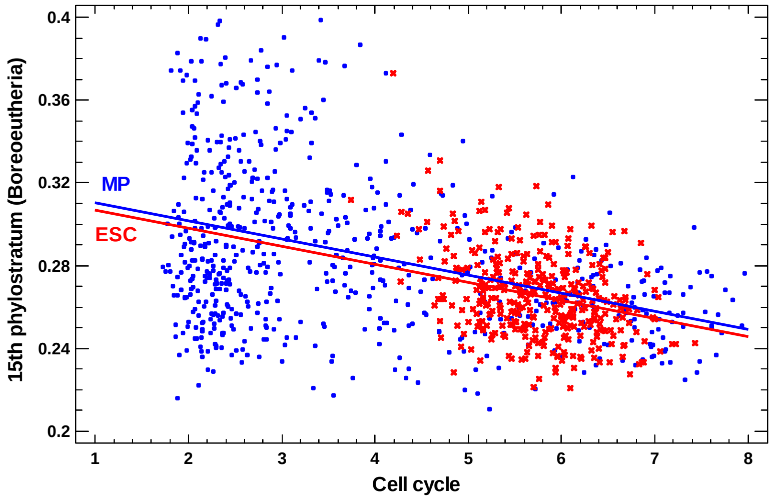

Figure S15. Regression lines of expression of genes belonging to 15th phylostratum (Boreoeutheria) on expression of cell-cycle signature in the single-cell transcriptomes of multipotent progenitors (MP) (blue) and pluripotent embryonic stem cells (ESC) (red). For difference between intercepts: $p>0.1$.

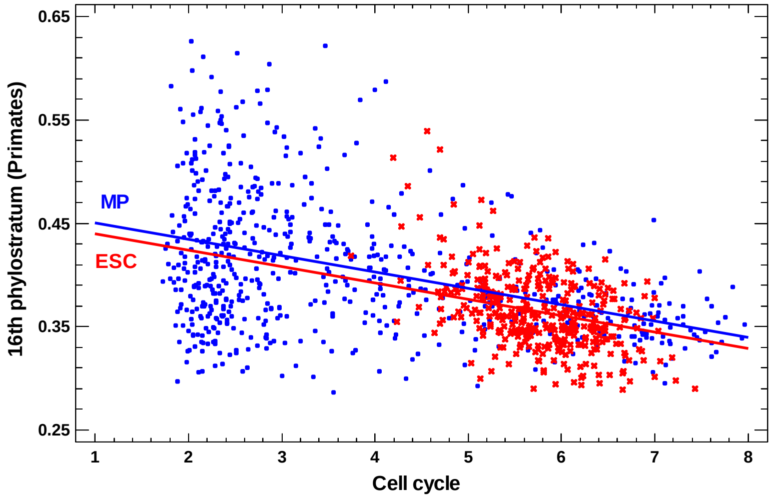

Figure S16. Regression lines of expression of genes belonging to 16th phylostratum (Primates) on expression of cell-cycle signature in the single-cell transcriptomes of multipotent progenitors (MP) (blue) and pluripotent embryonic stem cells (ESC) (red). For difference between intercepts: $p<0.05$.

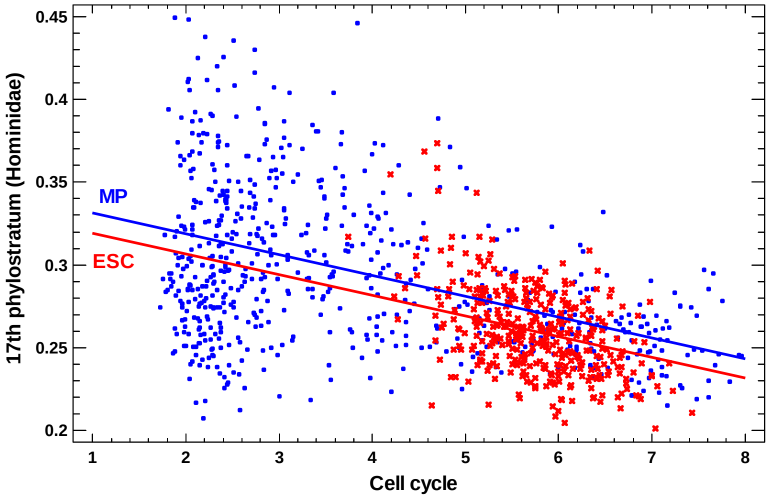

Figure S17. Regression lines of expression of genes belonging to 17th phylostratum (Hominidae) on expression of cell-cycle signature in the single-cell transcriptomes of multipotent progenitors (MP) (blue) and pluripotent embryonic stem cells (ESC) (red). For difference between intercepts: $p<10^{-3}$.
