# Supplementary material for: Cellular Biogenetic Law and Its Distortion by Protein Interactions: A Possible Unified Framework for Cancer Biology and Regenerative Medicine
Source: Int J Mol Sci. 2022 Sep 29;23(19):11486. doi: 10.3390/ijms231911486 (PMC9570048; doi:10.3390/ijms231911486)
Supplement: Supplementary file 1 [file ijms-23-11486-s001.zip › SupFigs_S18-S22.pdf]

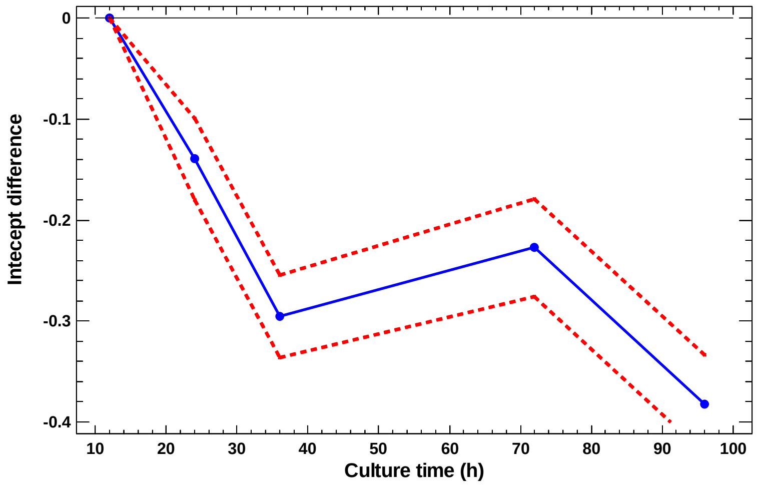

Figure S18. Differences in the intercepts between the regression lines of expression of UC genes (1-2 phylostrata) on cell-cycle signature in the single-cell transcriptomes of ESC cultured for 12-96 h (GSE75748, 'time course' dataset), with confidence intervals (p=0.05). The intercept for ESC cultured for 12 h is taken for zero. The step-wise downregulation of UC genes with the increase of culturing time can be seen (with the plateau at 36-72 h).

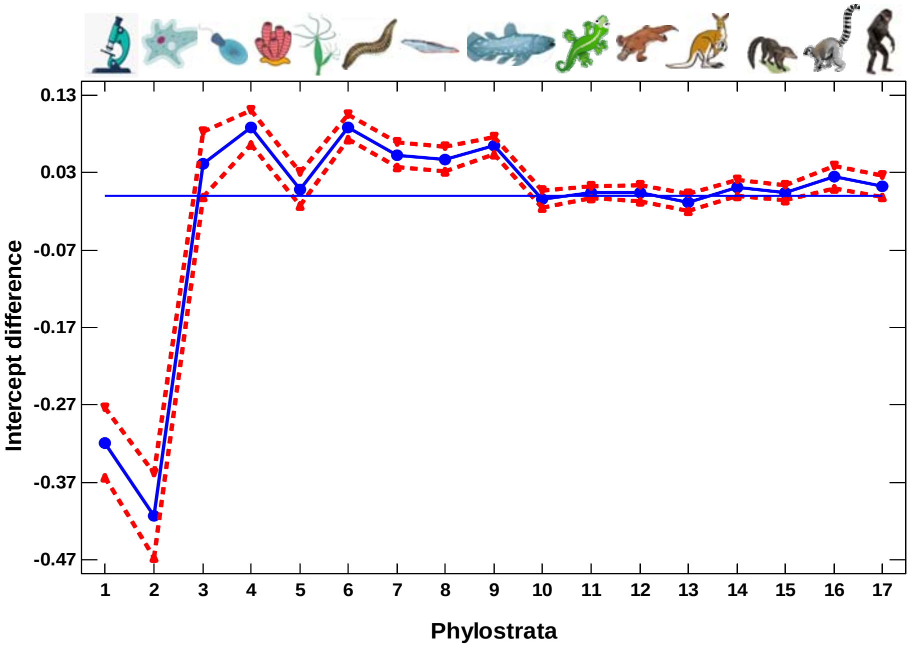

Figure S19. Evolutionary profiles: the differences in intercepts between the regression lines of expression of genes belonging to different phylostrata on cell-cycle signature in the single-cell transcriptomes of ESC cultured for 96 h vs. 12 h (GSE75748, 'time course' dataset), with confidence intervals (*p*=0.05).

Phylostrata: 1—cellular organisms (Prokaryota); 2—Eukaryota; 3—Opisthokonta; 4—Metazoa; 5—Eumetazoa; 6—Bilateria; 7—Chordata; 8—Vertebrata; 9—Euteleostomi; 10—Tetrapoda; 11—Amniota; 12—Mammalia; 13—Theria; 14—Eutheria; 15—Boreoeutheria; 16—Primates; 17—Hominidae.

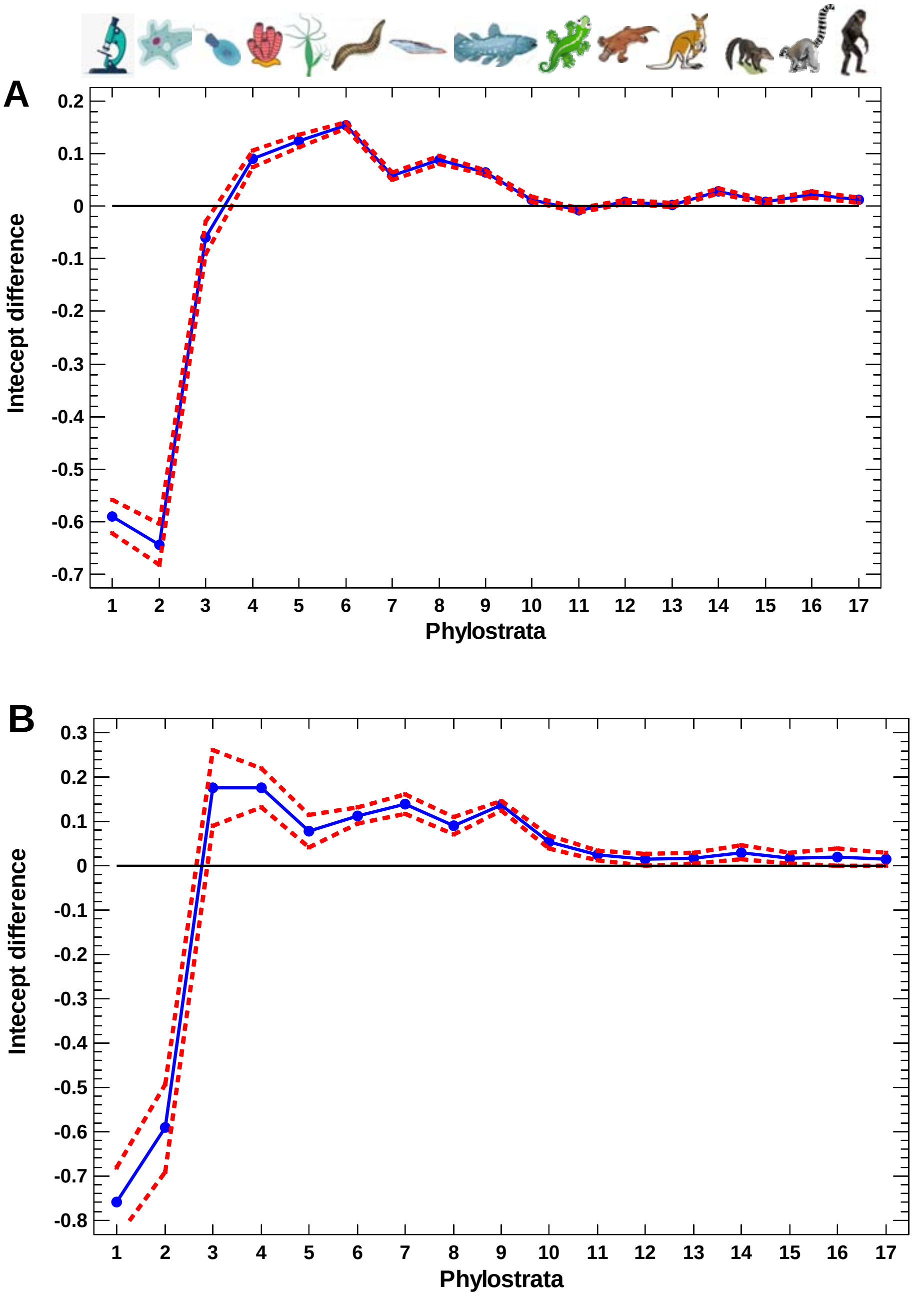

Figure S20. Evolutionary profiles: the differences in intercepts between the regression lines of expression of genes belonging to different phylostrata on cell-cycle signature in the single-cell transcriptomes for different cell types (with confidence intervals, p=0.05).

A -- neural progenitors (NPC) vs. ESC (GSE75748, 'cell type' dataset).

B -- endothelial cells, mesoderm derivatives (EC) vs. ESC (GSE75748, 'cell type' dataset).

Phylostrata: 1—cellular organisms (Prokaryota); 2—Eukaryota; 3—Opisthokonta; 4—Metazoa; 5—Eumetazoa; 6—Bilateria; 7—Chordata; 8—Vertebrata; 9—Euteleostomi; 10—Tetrapoda; 11—Amniota; 12—Mammalia; 13—Theria; 14—Eutheria; 15—Boreoeutheria; 16—Primates; 17—Hominidae.

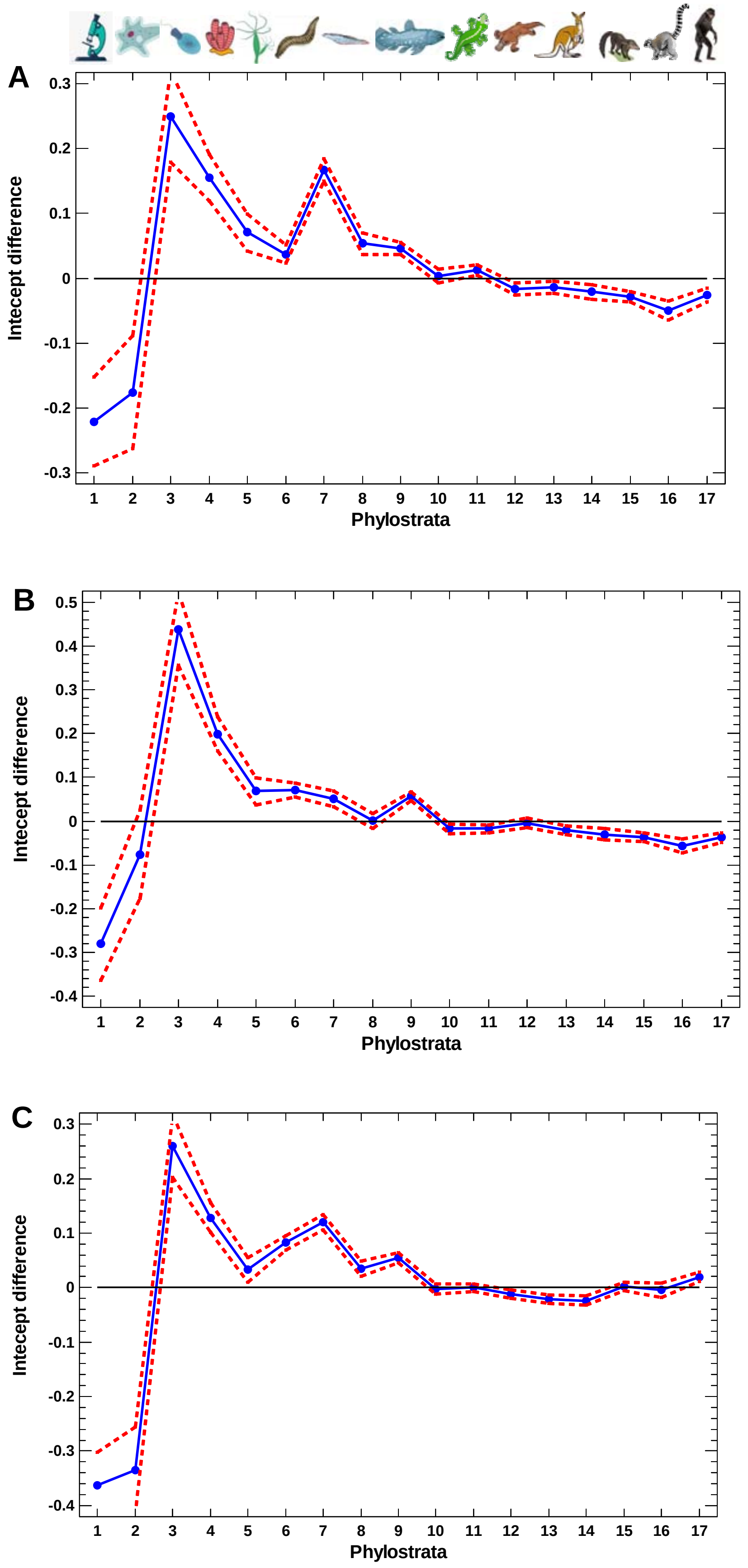
A
B
C
Intecept difference
Phylostrata
0.5
0.4
0.3
0.2
0.1
0
-0.1
-0.2
-0.3
-0.4
1 2 3 4 5 6 7 8 9 10 11 12 13 14 15 16 17

Figure S21. Evolutionary profiles: the differences in intercepts between the regression lines of expression of genes belonging to different phylostrata on cell-cycle signature in the single-cell transcriptomes for different cell types (with confidence intervals, *p*=0.05).
A -- foreskin fibroblasts (HFF) compared with ESC (GSE75748, 'cell type' dataset).
B -- trophoblast-like cells, extraembryonic derivatives (TB) compared with ESC (GSE75748, 'cell type' dataset).
C -- endoderm derivatives (DEC) compared with ESC (GSE75748, 'cell type' dataset).
Phylostrata: 1—cellular organisms (Prokaryota); 2—Eukaryota; 3—Opisthokonta; 4—Metazoa; 5—Eumetazoa; 6—Bilateria; 7—Chordata; 8—Vertebrata; 9—Euteleostomi; 10—Tetrapoda; 11—Amniota; 12—Mammalia; 13—Theria; 14—Eutheria; 15—Boreoeutheria; 16—Primates; 17—Hominidae.

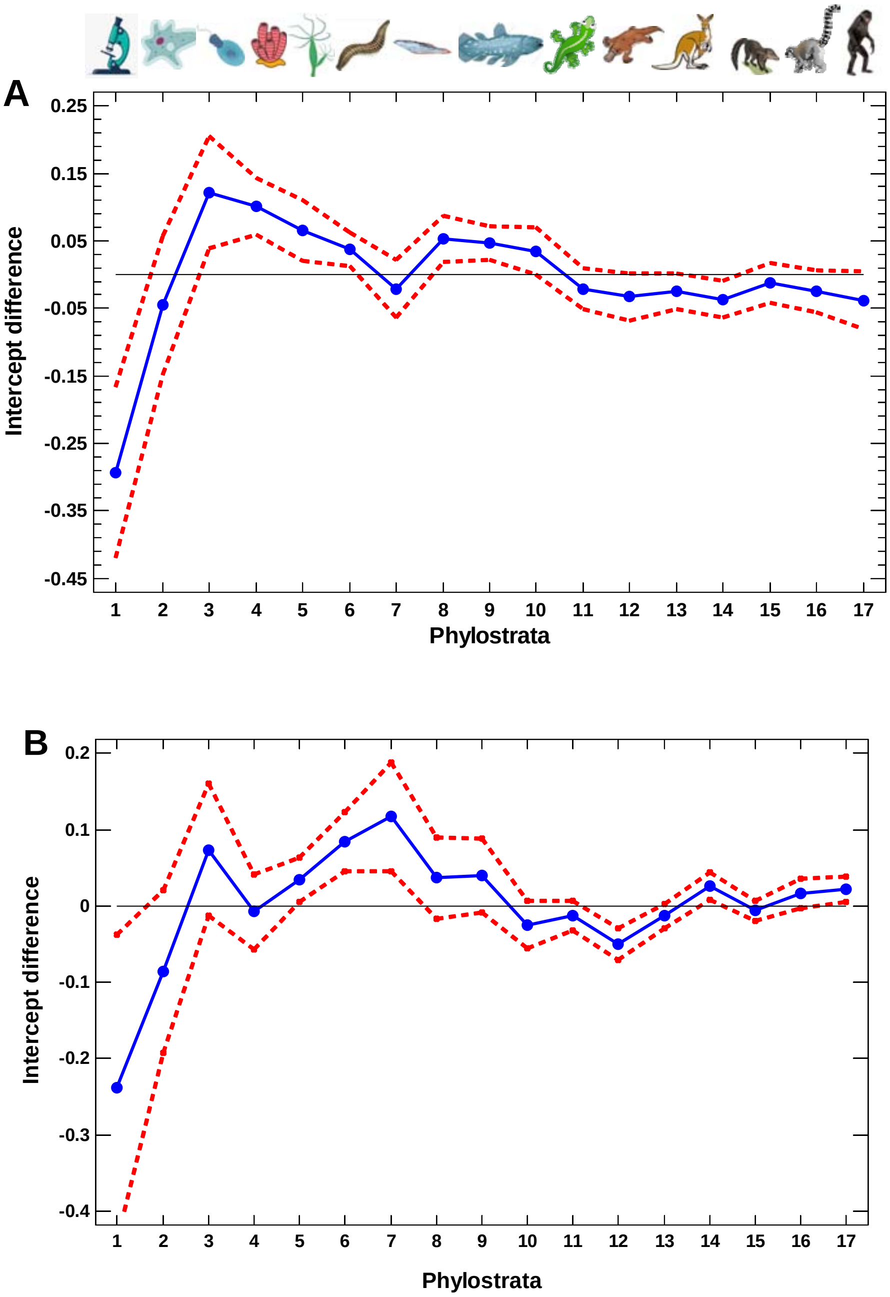

Figure S22. Evolutionary profiles: the differences in intercepts between the regression lines of expression of genes belonging to different phylostrata on cell-cycle signature in the single-cell transcriptomes for different cell types (with confidence intervals, p=0.05).

A -- mature hepatocytes compared with iPSC (GSE81252).

B -- white adipocytes compared with iPSC (GSE90749).

Phylostrata: 1—cellular organisms (Prokaryota); 2—Eukaryota; 3—Opisthokonta; 4—Metazoa; 5—Eumetazoa; 6—Bilateria; 7—Chordata; 8—Vertebrata; 9—Euteleostomi; 10—Tetrapoda; 11—Amniota; 12—Mammalia; 13—Theria; 14—Eutheria; 15—Boreoeutheria; 16—Primates; 17—Hominidae.
